# Supplementary material for: Anatomical and biomechanical traits of broiler chickens across ontogeny. Part II. Body segment inertial properties and muscle architecture of the pelvic limb
Source: PeerJ. 2014 Jul 3;2:e473. doi: 10.7717/peerj.473 (PMC4103074; doi:10.7717/peerj.473)
Supplement: Supplemental Information 1 — The material provided are all of the data used to formulate the results and conclusions of this study. Please note that numbers within tables have been rounded to 2–3 decimal places where necessary. We provide pelvic limb bone dimensions (Tables 1 and 4), pelvic limb segment inertial properties data (Table 2) and whole body CoM position data (Table 3). We also provide muscle architecture data (Table 5), including muscle mass, length, fascicle length, pennation angle and physiological cross-sectional area across ontogeny. [file peerj-02-473-s001.docx]

**Table 1 – Pelvic limb bone dimensions data**

| **Age Group** | **Bird Number** | **Total leg length** | **Bone lengths (% total leg length)** | | |
| --- | --- | --- | --- | --- | --- |
| **(days)** |  | **(cm)** | **Femur** | **Tibia** | **Tarsometatarsus** |
| 1 | 1 | 6.9 | 30.4 | 40.6 | 29.0 |
| 1 | 2 | 7.1 | 29.6 | 42.3 | 28.2 |
| 1 | 3 | 6.8 | 29.4 | 45.6 | 25.0 |
| 1 | 4 | 7.5 | 30.7 | 40.0 | 29.3 |
| 1 | 5 | 7.7 | 29.9 | 42.9 | 27.3 |
| 1 | 6 | 7.9 | 30.4 | 41.8 | 27.9 |
| 1 | 7 | 7.9 | 30.4 | 40.5 | 29.1 |
| 1 | 8 | 7.7 | 31.2 | 41.6 | 27.3 |
| 1 | 9 | 8.1 | 30.9 | 39.5 | 29.6 |
| 1 | 10 | 7.6 | 31.6 | 40.8 | 27.6 |
| 14 | 1 | 15.1 | 29.1 | 41.7 | 29.1 |
| 14 | 2 | 16.5 | 27.9 | 43.0 | 29.1 |
| 14 | 3 | 15.7 | 29.3 | 42.0 | 28.7 |
| 14 | 4 | 15.5 | 27.8 | 42.4 | 29.8 |
| 14 | 5 | 15.8 | 28.5 | 41.8 | 29.8 |
| 14 | 6 | 15.4 | 29.2 | 40.9 | 29.9 |
| 14 | 7 | 16.4 | 29.3 | 41.5 | 29.3 |
| 14 | 8 | 15.3 | 29.4 | 40.5 | 30.1 |
| 14 | 9 | 15.2 | 29.0 | 41.5 | 29.6 |
| 14 | 10 | 14.3 | 31.5 | 39.9 | 28.7 |
| 14 | 11 | 14.8 | 31.1 | 40.5 | 28.4 |
| 14 | 12 | 14.6 | 29.5 | 40.4 | 30.1 |
| 14 | 13 | 13.3 | 30.8 | 40.6 | 28.6 |
| 14 | 14 | 13.5 | 30.4 | 40.7 | 28.9 |
| 14 | 15 | 13.9 | 30.2 | 40.3 | 29.5 |
| 14 | 16 | 14.0 | 31.4 | 37.9 | 30.7 |
| 14 | 17 | 13.8 | 31.2 | 39.9 | 29.0 |
| 14 | 18 | 15.3 | 30.1 | 40.5 | 29.4 |
| 14 | 19 | 14.2 | 32.4 | 41.6 | 26.1 |
| 28 | 1 | 19.8 | 27.6 | 41.52 | 30.9 |
| 28 | 2 | 21.6 | 28.7 | 40.28 | 31.0 |
| 28 | 3 | 21.4 | 28.0 | 42.06 | 29.9 |
| 28 | 4 | 20.9 | 29.5 | 41.73 | 28.8 |
| 28 | 5 | 19.8 | 29.3 | 41.41 | 29.9 |
| 28 | 6 | 20.4 | 28.5 | 41.03 | 30.5 |
| 28 | 7 | 21.5 | 28.8 | 40.93 | 30.2 |
| 28 | 8 | 20.9 | 29.2 | 40.67 | 30.1 |
| 28 | 9 | 19.3 | 28.5 | 41.45 | 30.1 |
| 28 | 10 | 23.1 | 28.1 | 39.83 | 32.0 |

**Table 1 cont.**

| **Age Group** | **Bird Number** | **Total leg length** | **Bone lengths (% total leg length)** | | |
| --- | --- | --- | --- | --- | --- |
| **(days)** |  | **(cm)** | **Femur** | **Tibia** | **Tarsometatarsus** |
| 28 | 11 | 20.4 | 29.4 | 41.6 | 28.9 |
| 28 | 12 | 21.0 | 29.0 | 41.4 | 29.5 |
| 28 | 13 | 23.2 | 31.0 | 40.9 | 28.0 |
| 28 | 14 | 21.6 | 31.0 | 36.5 | 32.4 |
| 28 | 15 | 22.1 | 28.9 | 40.7 | 30.3 |
| 28 | 16 | 24.8 | 30.6 | 38.3 | 31.0 |
| 28 | 17 | 22.1 | 31.2 | 37.5 | 31.2 |
| 28 | 18 | 21.0 | 30.4 | 40.0 | 29.5 |
| 28 | 19 | 22.1 | 29.4 | 39.8 | 30.7 |
| 42 | 1 | 28.3 | 29.3 | 41.7 | 28.9 |
| 42 | 2 | 28.0 | 27.9 | 43.1 | 28.9 |
| 42 | 3 | 27.8 | 29.5 | 41.3 | 29.1 |
| 42 | 4 | 28.9 | 28.7 | 41.8 | 29.4 |
| 42 | 5 | 29.7 | 28.9 | 42.4 | 28.6 |
| 42 | 6 | 28.5 | 29.4 | 42.4 | 28.0 |
| 42 | 7 | 29.2 | 29.4 | 41.9 | 28.6 |
| 42 | 8 | 27.8 | 26.6 | 43.8 | 29.5 |
| 42 | 9 | 29.1 | 27.5 | 43.3 | 29.0 |
| 42 | 10 | 28.8 | 28.8 | 42.0 | 29.1 |
| 42 | 11 | 28.5 | 28.1 | 42.8 | 29.0 |
| 42 | 12 | 25.5 | 28.2 | 43.1 | 28.6 |
| 42 | 13 | 24.2 | 31.8 | 39.2 | 28.9 |
| 42 | 14 | 23.5 | 29.7 | 42.5 | 27.6 |
| 42 | 15 | 25.0 | 29.2 | 42.8 | 28.0 |
| 42 | 16 | 23.7 | 29.9 | 38.8 | 31.2 |
| 42 | 17 | 23.2 | 30.1 | 39.6 | 30.1 |
| 42 | 18 | 27.1 | 28.4 | 42.4 | 29.1 |
| 42 | 19 | 24.9 | 28.9 | 42.1 | 28.9 |

**Table 2 -** **Pelvic limb segment inertial properties data**

| Age Group | Segment | Bird Number | Segment Mass | CoM Position | Radius of gyration  (% segment length) | | |
| --- | --- | --- | --- | --- | --- | --- | --- |
| (days) |  |  | (% body mass) | (% segment length) | x | y | z |
| 14 | Trunk | 1 | 85.37 | 16.41 | 23.5 | 40.8 | 37.6 |
| 14 | Trunk | 2 | 83.50 | 17.69 | 24.1 | 41.4 | 37.9 |
| 14 | Trunk | 3 | 82.16 | 22.45 | 21.7 | 40.8 | 38.7 |
| 14 | Trunk | 4 | 77.09 | 21.09 | 24.2 | 41.5 | 36.6 |
| 14 | Trunk | 5 | 80.21 | 19.73 | 22.7 | 40.9 | 38.2 |
| 14 | Thigh | 1 | 4.68 | 43.05 | 39.2 | 46.3 | 46.4 |
| 14 | Thigh | 2 | 3.01 | 44.78 | 41.0 | 31.1 | 33.2 |
| 14 | Thigh | 3 | 6.48 | 48.77 |  | 46.8 | 39.5 |
| 14 | Thigh | 4 | 6.50 | 37.03 | 57.0 | 50.0 | 61.6 |
| 14 | Thigh | 5 | 5.28 | 44.64 | 51.5 | 46.2 | 67.5 |
| 14 | Drumstick | 1 | 3.92 | 36.70 | 44.8 | 47.7 | 59.6 |
| 14 | Drumstick | 2 | 3.59 | 4.54 | 59.9 | 61.4 | 27.7 |
| 14 | Drumstick | 3 | 4.32 | 35.53 | 48.7 | 51.0 | 27.1 |
| 14 | Drumstick | 4 | 3.76 | 37.37 | 47.1 | 49.2 | 22.9 |
| 14 | Drumstick | 5 | 3.67 | 36.09 | 44.7 | 46.0 | 21.0 |
| 14 | Shank | 1 | 0.91 | 17.91 | 52.3 | 52.5 | 17.9 |
| 14 | Shank | 2 | 0.92 | 43.51 | 49.7 | 49.9 | 18.2 |
| 14 | Shank | 3 | 1.07 | 40.15 | 51.2 | 51.3 | 18.2 |
| 14 | Shank | 4 | 0.92 | 38.01 | 50.0 | 50.2 | 18.2 |
| 14 | Shank | 5 | 1.07 | 41.03 | 49.6 | 50.0 | 17.7 |
| 14 | Foot | 1 | 0.63 | 35.07 | 33.0 | 34.4 | 21.9 |
| 14 | Foot | 2 | 0.62 | 39.48 | 35.3 | 33.7 | 21.7 |
| 14 | Foot | 3 | 0.68 | 45.23 | 35.0 | 33.7 | 23.5 |
| 14 | Foot | 4 | 0.62 | 34.44 | 34.4 | 37.2 | 25.0 |
| 14 | Foot | 5 | 0.60 | 38.87 | 33.9 | 37.2 | 24.2 |
| 28 | Trunk | 1 | 80.08 | 15.72 | 24.1 | 38.1 | 36.1 |
| 28 | Trunk | 2 | 78.40 | 25.96 | 24.3 | 38.5 | 34.6 |
| 28 | Trunk | 3 | 75.22 | 18.56 | 22.6 | 41.6 | 39.2 |
| 28 | Trunk | 4 | 78.83 | 22.91 | 23.3 | 40.1 | 37.2 |
| 28 | Trunk | 5 | 77.39 | 17.11 | 25.8 | 41.7 | 38.2 |
| 28 | Thigh | 1 | 5.63 | 37.23 | 53.2 | 40.4 | 60.2 |
| 28 | Thigh | 2 | 4.53 | 40.17 | 39.2 | 33.8 | 45.1 |
| 28 | Thigh | 3 | 5.49 | 36.38 | 48.6 | 45.5 | 61.4 |
| 28 | Thigh | 4 | 5.15 | 40.41 | 45.7 | 41.1 | 52.4 |
| 28 | Thigh | 5 | 5.30 | 36.45 | 54.7 | 42.0 | 41.5 |
| 28 | Drumstick | 1 | 4.26 | 31.40 | 36.7 | 39.0 | 22.3 |
| 28 | Drumstick | 2 | 4.23 | 34.44 | 45.4 | 47.9 | 23.3 |
| 28 | Drumstick | 3 | 4.25 | 35.87 | 44.6 | 46.0 | 22.6 |
| 28 | Drumstick | 4 | 5.15 | 30.73 | 33.0 | 38.0 | 29.7 |
| 28 | Drumstick | 5 | 5.30 | 30.99 | 39.6 | 30.0 | 30.3 |

| Age Group | Segment | Bird Number | Segment Mass | CoM Position | Radius of gyration  (% segment length) | | |
| --- | --- | --- | --- | --- | --- | --- | --- |
| (days) |  |  | (% body mass) | (% segment length) | x | y | z |
| 28 | Shank | 1 | 0.84 | 20.26 | 48.8 | 49.1 | 16.2 |
| 28 | Shank | 2 | 1.17 | 19.56 | 46.7 | 47.0 | 17.0 |
| 28 | Shank | 3 | 1.01 | 19.69 | 50.0 | 50.2 | 16.8 |
| 28 | Shank | 4 | 0.99 | 19.58 | 53.0 | 53.9 | 19.2 |
| 28 | Shank | 5 | 1.08 | 7.42 | 50.4 | 50.7 | 16.5 |
| 28 | Foot | 1 | 0.54 | 22.75 | 28.0 | 19.0 | 28.9 |
| 28 | Foot | 2 | 0.71 | 30.21 | 29.6 | 17.0 | 28.8 |
| 28 | Foot | 3 | 0.66 | 22.99 | 30.7 | 17.3 | 30.3 |
| 28 | Foot | 4 | 0.56 | 40.39 | 33.6 | 32.1 | 20.1 |
| 28 | Foot | 5 | 0.59 | 25.87 | 32.8 | 20.2 | 31.7 |
| 42 | Trunk | 1 | 75.72 | 15.33 | 47.4 | 35.5 | 37.7 |
| 42 | Trunk | 2 | 75.09 | 13.20 | 44.9 | 35.6 | 35.8 |
| 42 | Trunk | 3 | 74.82 | 13.53 | 25.5 | 38.8 | 33.8 |
| 42 | Trunk | 4 | 71.63 | 14.63 | 22.9 |  | 33.1 |
| 42 | Trunk | 5 | 75.59 | 19.80 | 25.2 | 38.2 | 34.2 |
| 42 | Thigh | 1 | 8.22 | 29.44 | 39.3 | 43.3 | 54.0 |
| 42 | Thigh | 2 | 7.55 | 26.65 | 57.7 | 48.4 | 60.2 |
| 42 | Thigh | 3 | 7.80 | 30.45 | 42.1 | 37.0 | 42.4 |
| 42 | Thigh | 4 | 9.95 | 25.74 | 45.4 | 40.0 | 48.1 |
| 42 | Thigh | 5 | 7.51 | 31.25 | 46.2 | 43.6 | 56.7 |
| 42 | Drumstick | 1 | 5.51 | 28.85 | 38.7 | 40.0 | 22.6 |
| 42 | Drumstick | 2 | 5.55 | 26.85 | 46.2 | 44.3 | 37.2 |
| 42 | Drumstick | 3 | 5.57 | 20.44 | 33.1 | 33.7 | 17.3 |
| 42 | Drumstick | 4 | 5.82 | 25.59 | 38.4 | 39.9 | 23.6 |
| 42 | Drumstick | 5 | 5.86 | 19.05 | 13.3 | 13.4 | 4.9 |
| 42 | Shank | 1 | 1.13 | 22.04 | 35.7 | 36.0 | 13.3 |
| 42 | Shank | 2 | 1.13 | 26.81 | 43.2 | 38.8 | 15.7 |
| 42 | Shank | 3 | 1.10 | 26.43 | 32.6 | 32.8 | 12.5 |
| 42 | Shank | 4 | 1.54 | 11.89 | 38.2 | 38.5 | 14.4 |
| 42 | Shank | 5 | 1.23 | 25.57 | 40.2 | 39.5 | 60.2 |
| 42 | Foot | 1 | 0.91 | 46.84 | 36.2 | 33.1 | 20.9 |
| 42 | Foot | 2 | 0.74 | 51.51 | 67.2 | 25.6 | 67.7 |
| 42 | Foot | 3 | 1.10 | 44.90 | 29.9 | 32.4 | 23.3 |
| 42 | Foot | 4 | 0.89 | 67.53 | 33.0 | 32.8 | 19.1 |
| 42 | Foot | 5 | 0.92 | 48.47 | 26.9 | 25.6 | 17.3 |

**Table 2 cont.**

**Table 3 – Whole Body Centre of Mass Position Data**

| Age Group | Bird Number | Centre of mass position  (% femur length) | |
| --- | --- | --- | --- |
| (days) |  | Cranial – caudal | Dorsal-ventral |
| 14 | 1 | 88.6 | 73.4 |
| 14 | 2 | 92.2 | 81.6 |
| 14 | 3 | 103.3 | 106.1 |
| 14 | 4 | 74.1 | 98.7 |
| 14 | 5 | 94.9 | 89.3 |
| 28 | 1 | 72.3 | 17.5 |
| 28 | 2 | 66.3 | 74.3 |
| 28 | 3 | 66.3 | 44.9 |
| 28 | 4 | 74.4 | 65.9 |
| 28 | 5 | 62.2 | 36.5 |
| 42 | 1 | 78.0 | 7.4 |
| 42 | 2 | 97.2 | 11.0 |
| 42 | 3 | 70.6 | 27.7 |
| 42 | 4 | 68.7 | 42.9 |
| 42 | 5 | 68.4 | 52.1 |

**Table 4 – Bone Scaling**

| Age Group | Bird Number | Body Mass | Femur | Tibia | TMT |
| --- | --- | --- | --- | --- | --- |
| (days) |  | (kg) | (cm) | (cm) | (cm) |
| 1 | 1 | 0.044 | 2.1 | 2.8 | 2.0 |
| 1 | 2 | 0.045 | 2.1 | 3.0 | 2.0 |
| 1 | 3 | 0.041 | 2.0 | 3.1 | 1.7 |
| 1 | 4 | 0.038 | 2.3 | 3.0 | 2.2 |
| 1 | 5 | 0.043 | 2.3 | 3.3 | 2.1 |
| 1 | 6 | 0.048 | 2.4 | 3.3 | 2.2 |
| 1 | 7 | 0.041 | 2.4 | 3.2 | 2.3 |
| 1 | 8 | 0.048 | 2.4 | 3.2 | 2.1 |
| 1 | 9 | 0.045 | 2.5 | 3.2 | 2.4 |
| 1 | 10 | 0.045 | 2.4 | 3.1 | 2.1 |
| 14 | 1 | 0.620 | 4.4 | 6.3 | 4.4 |
| 14 | 2 | 0.700 | 4.6 | 7.1 | 4.8 |
| 14 | 3 | 0.660 | 4.6 | 6.6 | 4.5 |
| 14 | 4 | 0.540 | 4.3 | 6.6 | 4.6 |
| 14 | 6 | 0.600 | 4.5 | 6.6 | 4.7 |
| 14 | 7 | 0.540 | 4.5 | 6.3 | 4.6 |
| 14 | 8 | 0.580 | 4.8 | 6.8 | 4.8 |
| 14 | 9 | 0.560 | 4.5 | 6.2 | 4.6 |
| 14 | 10 | 0.480 | 4.4 | 6.3 | 4.5 |
| 14 | 11 | 0.440 | 4.5 | 5.7 | 4.1 |
| 14 | 12 | 0.451 | 4.6 | 6.0 | 4.2 |
| 14 | 13 | 0.394 | 4.3 | 5.9 | 4.4 |
| 14 | 14 | 0.434 | 4.1 | 5.4 | 3.8 |
| 14 | 15 | 0.436 | 4.1 | 5.5 | 3.9 |
| 14 | 16 | 0.441 | 4.2 | 5.6 | 4.1 |
| 14 | 17 | 0.382 | 4.4 | 5.3 | 4.3 |
| 14 | 18 | 0.446 | 4.3 | 5.5 | 4.0 |
| 14 | 19 | 0.424 | 4.6 | 6.2 | 4.5 |
| 14 | 20 | 0.459 | 4.6 | 5.9 | 3.7 |
| 28 | 1 | 1.320 | 5.5 | 8.2 | 6.1 |
| 28 | 2 | 1.420 | 6.2 | 8.7 | 6.7 |
| 28 | 3 | 1.380 | 6.0 | 9.0 | 6.4 |
| 28 | 4 | 1.580 | 6.2 | 8.7 | 6.0 |
| 28 | 5 | 1.300 | 5.8 | 8.2 | 5.8 |
| 28 | 6 | 1.460 | 5.8 | 8.4 | 6.2 |
| 28 | 7 | 1.500 | 6.2 | 8.8 | 6.5 |
| 28 | 8 | 1.380 | 6.1 | 8.5 | 6.3 |
| 28 | 9 | 1.340 | 5.5 | 8.0 | 5.8 |
| 28 | 10 | 1.425 | 6.5 | 9.2 | 7.4 |

**Table 4 cont.**

| Age Group | Bird Number | Body Mass | Femur | Tibia | TMT |
| --- | --- | --- | --- | --- | --- |
| (days) |  | (kg) | (cm) | (cm) | (cm) |
| 28 | 11 | 1.468 | 6.0 | 8.5 | 5.9 |
| 28 | 12 | 1.559 | 6.1 | 8.7 | 6.2 |
| 28 | 13 | 1.717 | 7.2 | 9.5 | 6.5 |
| 28 | 14 | 1.545 | 6.7 | 7.9 | 7.0 |
| 28 | 15 | 1.671 | 6.4 | 9.0 | 6.7 |
| 28 | 16 | 2.023 | 7.6 | 9.5 | 7.7 |
| 28 | 17 | 1.594 | 6.9 | 8.3 | 6.9 |
| 28 | 18 | 1.798 | 6.4 | 8.4 | 6.2 |
| 28 | 19 | 1.606 | 6.5 | 8.8 | 6.8 |
| 42 | 1 | 3.254 | 8.3 | 11.8 | 8.2 |
| 42 | 2 | 3.250 | 7.8 | 12.1 | 8.1 |
| 42 | 3 | 3.250 | 8.2 | 11.5 | 8.1 |
| 42 | 4 | 3.358 | 8.3 | 12.1 | 8.5 |
| 42 | 5 | 3.200 | 8.6 | 12.6 | 8.5 |
| 42 | 6 | 3.496 | 8.4 | 12.1 | 8.0 |
| 42 | 7 | 3.152 | 8.6 | 12.3 | 8.35 |
| 42 | 8 | 3.686 | 7.4 | 12.2 | 8.2 |
| 42 | 9 | 3.475 | 8.0 | 12.6 | 8.45 |
| 42 | 10 | 3.222 | 8.3 | 12.1 | 8.4 |
| 42 | 11 | 3.178 | 8.0 | 12.2 | 8.25 |
| 42 | 12 | 2.786 | 7.2 | 11.0 | 7.3 |
| 42 | 13 | 2.917 | 7.7 | 9.5 | 7.0 |
| 42 | 14 | 2.529 | 7.0 | 10.0 | 6.5 |
| 42 | 15 | 2.474 | 7.3 | 10.7 | 7.0 |
| 42 | 16 | 2.414 | 7.1 | 9.2 | 7.4 |
| 42 | 17 | 2.209 | 7.0 | 9.2 | 7.0 |
| 42 | 18 | 2.470 | 7.7 | 11.5 | 7.9 |
| 42 | 19 | 2.155 | 7.2 | 10.5 | 7.2 |

**Table 5 - Muscle Architecture Data (Mean ± SD)**

| **Age (days)** | **Muscle** | ***M*_m_ (g)** | ***M*_L_ (mm)** | ***F*_L_ (mm)** | **Angle (°)** | **PCSA (mm^2^)** |
| --- | --- | --- | --- | --- | --- | --- |
| **1** | *M. iliotibialis cranialis* (IC) | 0.15 ± 0.06 | 27.60 ± 4.09 | 23.24 ± 4.14 | - | 5.91 ± 2.58 |
|  | *M. iliotibialis lateralis* (IL) | 0.24 ± 0.05 | 28.90 ± 2.18 | 16.71 ± 3.78 | - | 14.16 ± 5.49 |
|  | *M. gastrocnemius pars lateralis* (GL) | 0.10 ± 0.01 | 21.50 ± 3.21 | 7.72 ± 2.03 | 26.91 ± 3.34 | 12.73 ± 3.66 |
|  | *M. gastrocnemius pars medialis* (GM) | 0.21 ± 0.05 | 25.80 ± 2.70 | 13.58 ± 2.72 | 25.98 ± 5.10 | 12.56 ± 3.88 |
|  | *M. fibularis longus* (FL) | 0.09 ± 0.02 | 21.40 ± 2.17 | 9.23 ± 3.08 | 26.74 ± 3.76 | 8.66 ± 3.47 |
|  | *M. iliotrochantericus caudalis* (ITC) | 0.10 ± 0.02 | 13.20 ± 1.69 | 6.36 ± 2.13 | 34.32 ± 4.06 | 15.29 ± 3.25 |
|  | *M. femorotibialis* (FMT) | 0.20 ± 0.04 | 18.89 ± 3.41 | 10.87 ± 2.30 | 27.75 | 22.07 |
|  | *M. iliofibularis* (ILFB) | 0.12 ± 0.02 | 22.50 ± 3.63 | 18.78 ± 2.18 | - | 6.03 ± 1.38 |
|  | *M. flexor cruris lateralis pelvica* (FCLP) | 0.10 ± 0.03 | 29.40 ± 2.50 | 22.34 ± 3.28 | - | 4.16 ± 0.78 |
|  | *M. flexor cruris* *medialis* (FCM) | 0.05 ± 0.04 | 18.00 ± 2.92 | 15.13 ± 2.36 | - | 2.17 ± 0.60 |
|  | *M. caudofemoralis pars caudalis* (CFC) | 0.01 ± 0.01 | 21.50 ± 2.56 | 16.53 ± 3.37 | - | 0.38 ± 0.28 |
|  | *M. tibialis cranialis caput femorale* (TCF) | 0.11 ± 0.03 | 21.80 ± 3.05 | 13.47 ± 1.84 | 20.60 | 7.40 |
|  | *M. puboischiofemoralis pars medialis* (PIFM) | 0.07 ± 0.03 | 16.00 ± 1.58 | 12.29 ± 1.84 | - | 5.43 ± 1.67 |
|  | *M. puboischiofemoralis pars lateralis* (PIFL) | 0.06 ± 0.02 | 17.89 ± 2.26 | 14.62 ± 2.36 | - | 3.72 ± 1.42 |

**Table 5 cont.**

| **Age (days)** | **Muscle** | ***M*_m_ (g)** | ***M*_L_ (mm)** | ***F*_L_ (mm)** | **Angle (°)** | **PCSA (mm^2^)** |
| --- | --- | --- | --- | --- | --- | --- |
| **13** | *M. iliotibialis cranialis* (IC) | 1.16 ± 0.26 | 54.10 ± 2.13 | 47.22 ± 2.90 | - | 23.32 ± 6.32 |
|  | *M. iliotibialis lateralis* (IL) | 3.14 ± 0.54 | 65.90 ± 2.73 | 42.80 ± 5.69 | - | 69.96 ± 12.42 |
|  | *M. gastrocnemius pars lateralis* (GL) | 1.35 ± 1.35 | 43.70 ± 3.37 | 13.38 ± 1.93 | 28.64 ± 3.50 | 83.70 ± 10.71 |
|  | *M. gastrocnemius pars medialis* (GM) | 2.37 ± 0.37 | 56.10 ± 6.08 | 22.78 ± 2.86 | 26.88 ± 4.07 | 88.61 ± 18.91 |
|  | *M. fibularis longus* (FL) | 1.21 ± 0.28 | 45.40 ± 5.10 | 12.84 ± 2.76 | 26.16 ± 3.37 | 81.99 ± 22.91 |
|  | *M. iliotrochantericus caudalis* (ITC) | 1.35 ± 0.24 | 25.30 ± 1.49 | 11.74 ± 1.89 | 34.12 ± 3.76 | 91.28 ± 20.49 |
|  | *M. femorotibialis* (FMT) | 2.00 ± 0.25 | 36.20 ± 4.16 | 21.08 ± 4.84 | 34.67 ± 3.26 | 107.21 ± 21.78 |
|  | *M. iliofibularis* (ILFB) | 1.36 ± 0.23 | 46.40 ± 2.55 | 39.50 ± 3.61 | - | 32.73 ± 6.39 |
|  | *M. flexor cruris lateralis pelvica* (FCLP) | 1.58 ± 0.31 | 72.30 ± 12.10 | 47.83 ± 8.71 | - | 31.96 ± 8.62 |
|  | *M. flexor cruris* *medialis* (FCM) | 0.40 ± 0.09 | 37.10 ± 3.73 | 32.71 ± 2.80 | - | 11.50 ± 2.13 |
|  | *M. caudofemoralis pars caudalis* (CFC) | 0.14 ± 0.06 | 50.78 ± 11.40 | 39.83 ± 10.61 | - | 3.17 ± 1.45 |
|  | *M. tibialis cranialis caput femorale* (TCF) | 1.07 ± 0.25 | 40.70 ± 4.57 | 24.52 ± 3.68 | 26.55 ± 6.34 | 43.53 ± 16.39 |
|  | *M. puboischiofemoralis pars medialis* (PIFM) | 0.63 ± 0.17 | 37.40 ± 5.64 | 24.88 ± 3.62 | - | 24.00 ± 6.65 |
|  | *M. puboischiofemoralis pars lateralis* (PIFL) | 0.55 ± 0.17 | 34.20 ± 4.13 | 29.06 ± 4.79 | - | 17.93 ± 4.81 |

**Table 5 cont.**

| **Age (days)** | **Muscle** | ***M*_m_ (g)** | ***M*_L_ (mm)** | ***F*_L_ (mm)** | **Angle (°)** | **PCSA (mm^2^)** |
| --- | --- | --- | --- | --- | --- | --- |
| ***29*** | *M. iliotibialis cranialis (IC)* | *4.06 ± 1.03* | *82.80 ± 4.49* | *65.60 ± 15.59* | *-* | *58.95 ± 8.41* |
|  | *M. iliotibialis lateralis (IL)* | *12.68 ± 1.07* | *88.92 ± 19.40* | *63.16 ± 9.41* | *-* | *192.07 ± 27.27* |
|  | *M. gastrocnemius pars lateralis (GL)* | *5.56 ± 0.80* | *61.20 ± 6.46* | *29.44 ± 3.04* | *32.76 ± 5.42* | *233.54 ± 64.04* |
|  | *M. gastrocnemius pars medialis (GM)* | *9.21 ± 1.88* | *84.60 ± 9.69* | *26.48 ± 2.36* | *28.64 ± 3.76* | *292.38 ± 84.79* |
|  | *M. fibularis longus (FL)* | *4.38 ± 0.89* | *64.20 ± 2.39* | *22.60 ± 7.47* | *26.16 ± 4.99* | *179.83 ± 80.41* |
|  | *M. iliotrochantericus caudalis (ITC)* | *5.37 ± 1.25* | *39.60 ± 2.07* | *17.32 ± 1.11* | *34.36 ± 3.20* | *241.51 ± 56.89* |
|  | *M. femorotibialis (FMT)* | *8.00 ± 1.97* | *53.60 ± 7.89* | *30.64 ± 9.95* | *-* | *-* |
|  | *M. iliofibularis (ILFB)* | *5.85 ± 1.04* | *68.60 ± 2.97* | *55.60 ± 5.13* | *-* | *99.32 ± 16.04* |
|  | *M. flexor cruris lateralis pelvica (FCLP)* | *8.10 ± 1.71* | *107.60 ± 12.50* | *72.12 ± 6.75* | *-* | *106.97 ± 24.55* |
|  | *M. flexor cruris medialis (FCM)* | *1.70 ± 0.36* | *54.20 ± 3.96* | *45.48 ± 4.20* | *-* | *35.35 ± 7.28* |
|  | *M. caudofemoralis pars caudalis (CFC)* | *0.54 ± 0.13* | *64.00 ± 4.95* | *55.48 ± 3.74* | *-* | *9.16 ± 1.75* |
|  | *M. tibialis cranialis caput femorale (TCF)* | *3.94 ± 1.37* | *68.60 ± 7.80* | *36.31 ± 13.22* |  | *101.38 ± 33.35* |
|  | *M. puboischiofemoralis pars medialis (PIFM)* | *2.99 ± 0.58* | *52.80 ± 3.70* | *31.32 ± 8.30* | *-* | *92.11 ± 15.78* |
|  | *M. puboischiofemoralis pars lateralis (PIFL)* | *1.80 ± 0.19* | *42.40 ± 4.04* | *40.60 ± 6.43* | *-* | *42.29 ± 3.24* |

**Table 5 cont.**

| **Age (days)** | **Muscle** | ***M*_m_ (g)** | ***M*_L_ (mm)** | ***F*_L_ (mm)** | **Angle (°)** | **PCSA (mm^2^)** |
| --- | --- | --- | --- | --- | --- | --- |
| **32** | *M. iliotibialis cranialis* (IC) | 5.68 ± 0.28 | 90.20 ± 9.34 | 80.96 ± 9.17 | - | 66.91 ± 9.50 |
|  | *M. iliotibialis lateralis* (IL) | 15.19 ± 2.23 | 98.00 ± 2.35 | 70.84 ± 7.36 | - | 203.12 ± 30.71 |
|  | *M. gastrocnemius pars lateralis* (GL) | 6.22 ± 0.68 | 61.60 ± 2.30 | 20.04 ± 3.56 | 31.32 ± 3.25 | 253.04 ± 31.48 |
|  | *M. gastrocnemius pars medialis* (GM) | 11.70 ± 1.32 | 88.00 ± 7.04 | 26.60 ± 3.23 | 25.84 ± 4.27 | 374.07 ± 37.57 |
|  | *M. fibularis longus* (FL) | 6.13 ± 0.87 | 66.80 ± 4.15 | 13.76 ± 2.24 | 27.44 ± 5.23 | 375.34 ± 47.36 |
|  | *M. iliotrochantericus caudalis* (ITC) | 5.19 ± 0.56 | 35.20 ± 2.59 | 80.96 ± 1.53 | 35.20 ± 2.48 | 245.64 ± 21.76 |
|  | *M. femorotibialis* (FMT) | 6.91 ± 2.16 | 54.20 ± 5.67 | 31.24 ± 7.35 | - | - |
|  | *M. iliofibularis* (ILFB) | 7.21 ± 0.92 | 65.80 ± 8.61 | 58.28 ± 7.04 | - | 117.06 ± 11.21 |
|  | *M. flexor cruris lateralis pelvica* (FCLP) | 9.51 ± 1.68 | 109.00 ± 11.40 | 74.64 ± 12.94 | - | 123.35 ± 29.98 |
|  | *M. flexor cruris* medialis (FCM) | 1.74 ± 0.39 | 54.60 ± 10.30 | 49.24 ± 7.65 | - | 33.30 ± 5.39 |
|  | *M. caudofemoralis pars caudalis* (CFC) | 0.64 ± 0.14 | 76.60 ± 9.21 | 62.16 ± 11.87 | - | 9.75 ± 1.49 |
|  | *M. tibialis cranialis caput femorale* (TCF) | 4.90 ± 0.91 | 69.20 ± 7.60 | 43.63 ± 12.83 | 24.50 | 155.23 |
|  | *M. puboischiofemoralis pars medialis* (PIFM) | 3.20 ± 0.38 | 54.20 ± 1.92 | 40.12 ± 3.99 | - | 75.66 ± 10.35 |
|  | *M. puboischiofemoralis pars lateralis* (PIFL) | 2.34 ± 0.38 | 50.80 ± 1.79 | 44.32 ± 2.21 | - | 49.77 ± 7.89 |

**Table 5 cont.**

| **Age (days)** | **Muscle** | ***M*_m_ (g)** | ***M*_L_ (mm)** | ***F*_L_ (mm)** | **Angle (°)** | **PCSA (mm^2^)** |
| --- | --- | --- | --- | --- | --- | --- |
| **40** | *M. iliotibialis cranialis* (IC) | 8.60 ± 1.61 | 108.57 ± 12.10 | 89.57 ± 7.23 | - | 90.55 ± 15.41 |
|  | *M. iliotibialis lateralis* (IL) | 22.57 ± 2.74 | 114.57 ± 9.43 | 76.69 ± 7.67 | - | 280.02 ± 44.31 |
|  | *M. gastrocnemius pars lateralis* (GL) | 9.76 ± 1.48 | 76.86 ± 6.62 | 25.03 ± 2.59 | 33.37 ± 4.20 | 309.46 ± 57.84 |
|  | *M. gastrocnemius pars medialis* (GM) | 16.91 ± 2.25 | 102.29 ± 11.80 | 32.11 ± 5.08 | 29.11 ± 4.84 | 437.34 ± 57.38 |
|  | *M. fibularis longus* (FL) | 9.51 ± 1.44 | 88.57 ± 12.60 | 18.03 ± 6.94 | 27.74 ± 4.19 | 484.71 ± 154.77 |
|  | *M. iliotrochantericus caudalis* (ITC) | 7.28 ± 1.14 | 42.57 ± 2.44 | 21.66 ± 2.97 | 35.29 ± 7.17 | 257.30 ± 29.78 |
|  | *M. femorotibialis* (FMT) | 12.73 ± 2.03 | 64.14 ± 9.41 | 34.11 ± 11.24 | 17.62 ± 16.89 | 380.34 ± 47.21 |
|  | *M. iliofibularis* (ILFB) | 10.10 ± 1.26 | 75.14 ± 6.09 | 61.57 ± 4.42 | - | 156.06 ± 27.12 |
|  | *M. flexor cruris lateralis pelvica* (FCLP) | 14.42 ± 0.92 | 125.00 ± 6.56 | 81.89 ± 15.90 | - | 171.16 ± 33.35 |
|  | *M. flexor cruris* medialis (FCM) | 3.30 ± 0.25 | 65.00 ± 11.70 | 53.80 ± 8.57 | - | 58.50 ± 5.30 |
|  | *M. caudofemoralis pars caudalis* (CFC) | 0.99 ± 0.23 | 85.00 ± 5.42 | 68.04 ± 6.10 | - | 13.66 ± 2.82 |
|  | *M. tibialis cranialis caput femorale* (TCF) | 7.56 ± 1.72 | 82.00 ± 10.60 | 35.31 ± 13.18 | 27.57 ± 5.05 | 205.71 ± 65.62 |
|  | *M. puboischiofemoralis pars medialis* (PIFM) | 4.82 ± 1.03 | 62.57 ± 7.87 | 45.14 ± 6.76 | - | 102.84 ± 27.33 |
|  | *M. puboischiofemoralis pars lateralis* (PIFL) | 3.58 ± 0.61 | 55.71 ± 5.44 | 48.54 ± 7.88 | - | 71.73 ± 19.38 |
